# Conventional MRI findings in hereditary degenerative ataxias: a pictorial review

**DOI:** 10.1007/s00234-021-02682-2

**Published:** 2021-03-17

**Authors:** Sirio Cocozza, Giuseppe Pontillo, Giovanna De Michele, Martina Di Stasi, Elvira Guerriero, Teresa Perillo, Chiara Pane, Anna De Rosa, Lorenzo Ugga, Arturo Brunetti

**Affiliations:** 1 Department of Advanced Biomedical Sciences, University of Naples “Federico II”, Via Pansini, 5, 80131 Naples, Italy; 2 Department of Electrical Engineering and Information Technology, University of Naples “Federico II”, Naples, Italy; 3 Department of Neurosciences and Reproductive and Odontostomatological Sciences, University of Naples “Federico II”, Naples, Italy

**Keywords:** Cerebellar ataxia, Magnetic resonance imaging, Conventional MRI

## Abstract

**Purpose:**

Cerebellar ataxias are a large and heterogeneous group of disorders. The evaluation of brain parenchyma via MRI plays a central role in the diagnostic assessment of these conditions, being mandatory to exclude the presence of other underlying causes in determining the clinical phenotype. Once these possible causes are ruled out, the diagnosis is usually researched in the wide range of hereditary or sporadic ataxias.

**Methods:**

We here propose a review of the main clinical and conventional imaging findings of the most common hereditary degenerative ataxias, to help neuroradiologists in the evaluation of these patients.

**Results:**

Hereditary degenerative ataxias are all usually characterized from a neuroimaging standpoint by the presence, in almost all cases, of cerebellar atrophy. Nevertheless, a proper assessment of imaging data, extending beyond the mere evaluation of cerebellar atrophy, evaluating also the pattern of volume loss as well as concomitant MRI signs, is crucial to achieve a proper diagnosis.

**Conclusion:**

The integration of typical neuroradiological characteristics, along with patient’s clinical history and laboratory data, could allow the neuroradiologist to identify some conditions and exclude others, addressing the neurologist to the more appropriate genetic testing.

## Introduction

Cerebellar ataxias are a large group of disorders characterized by various clinical presentations, ranging from the presence of a pure cerebellar phenotype to a heterogeneous combination of cerebellar signs along with extra-cerebellar symptoms [1]. Although still very challenging, different workflows have been proposed to help clinicians in identifying the cause of the clinical presentation and reach a proper diagnosis [2–4]. In all cases, the in vivo evaluation of brain parenchyma via MRI plays a pivotal role in the diagnostic assessment of cerebellar ataxias [5]. Indeed, it is mandatory to exclude that the observed cerebellar involvement could be due to structural damage secondary to non-degenerative conditions (e.g., stroke, neoplasm, metabolic or toxic disorders). Once these possible causes are ruled out in determining the cerebellar symptoms, the diagnosis is usually researched in the wide range of hereditary or sporadic ataxias, which are all usually characterized only by the presence of non-specific and sometimes overlapping imaging findings, with cerebellar atrophy being the least common denominator. Nevertheless, although challenging, a proper evaluation of imaging data and the integration of the patient’s clinical history could allow the neuroradiologist to identify some conditions and exclude others, addressing the neurologist to the more appropriate genetic testing.

Given this background, here, we propose a review of the main clinical and conventional imaging findings of the most common hereditary degenerative ataxias, to highlight the main features in these conditions. We will discuss degenerative ataxias according to their frequency and clinical relevance [6], while in this review, we will not cover the malformative conditions (e.g., ponto-cerebellar hypoplasias, some tubulinopathies, or certain dystroglycanopathies), given their different pathophysiology and imaging appearance.

## Autosomal dominant ataxias

### Spinocerebellar ataxia type 1

Spinocerebellar ataxia type 1 (SCA1) accounts for 6% of autosomal dominant cerebellar ataxias [7]. Affected individuals have 39 or more CAG trinucleotide repeats in the *ATXN1* gene, which encodes for the *Ataxin1* protein [8]. Onset is typically between the third and the fourth decades, even though childhood onset has been reported [9, 10]. The phenotype comprises a cerebellar syndrome with ataxia of gait, stance, and limbs, dysarthria, and oculomotor abnormalities. Pyramidal signs are common, but amyotrophy and sensory loss also occur [7]. The duration of disease from onset to death is 10 to 30 years in the adult-onset forms, whereas it is more rapid and severe in juvenile-onset forms [10].

Brain MRI typically shows olivo-ponto-cerebellar atrophy and white matter volume decrease, with a similar distribution but less severe than SCA2 [11, 12].

**Fig. 1:**
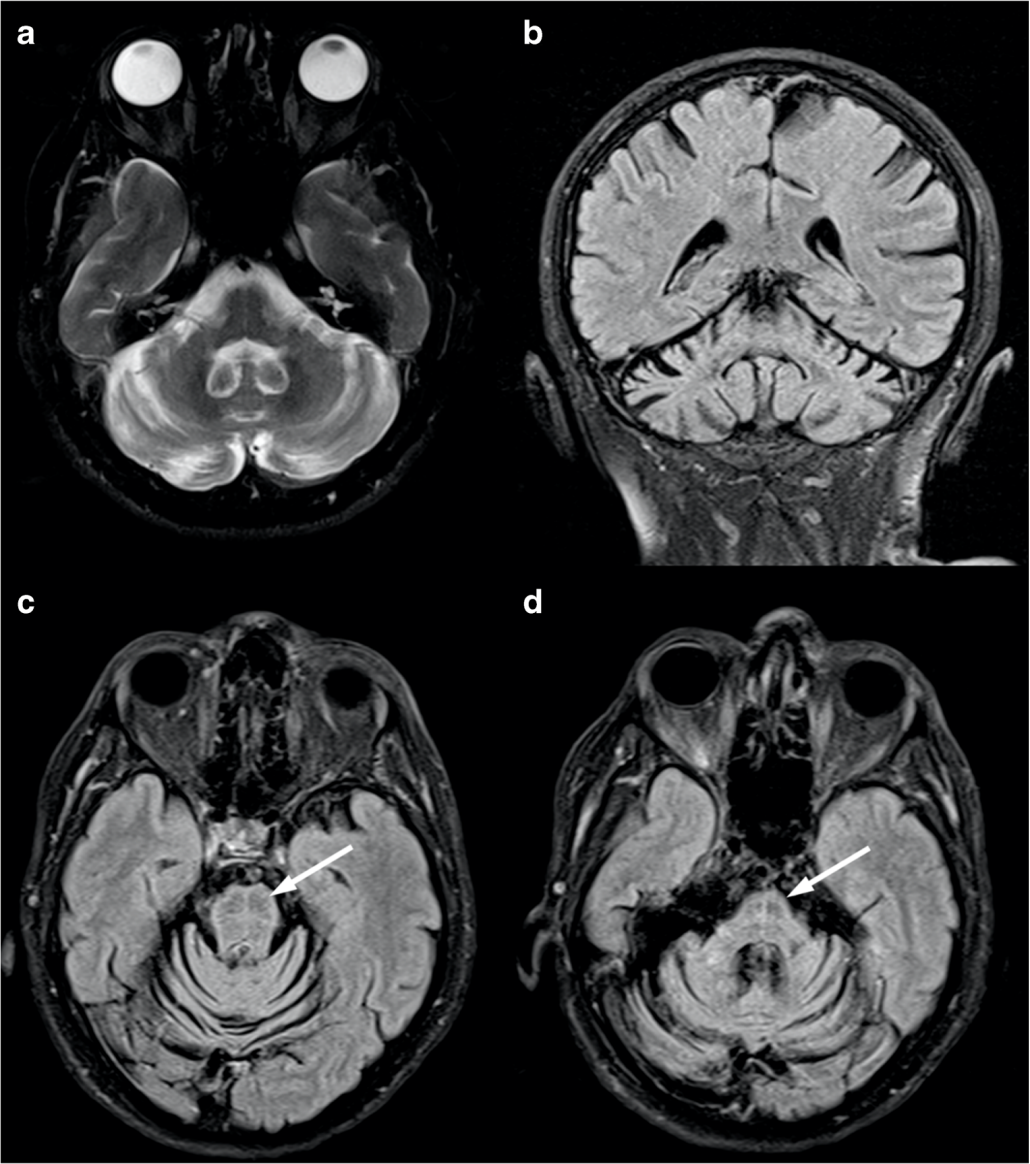
Brain MRI scan of a 69-year-old female SCA1 patient. Axial T2-weighted (**a**) and coronal FLAIR (**b**) images show a global cerebellar volume loss, along with brainstem atrophy (**c**–**d**) and the presence of the “hot cross bun” sign (arrows)

The presence of a midline T2-hyperintensity in the pontine base has been reported [13] as well as a cruciform pontine T2-hyperintensity, the “hot cross bun” sign, due to ponto-cerebellar fibers degeneration [14] (Fig. 1). Usually, the supratentorial compartment is relatively spared by the disease, with high-intensity areas on T2-weighted images in the frontal white matter only anecdotally reported [15]. Finally, spinal cord volume reduction might be present [12] and correlates with the SARA score, length of CAG expansion, and disease duration [16].

### Spinocerebellar ataxia type 2

Spinocerebellar ataxia type 2 (SCA2) is caused by an abnormal expansion of CAG repetition (>33) in the *ATXN2* gene, coding for *Ataxin2* protein [17], with an estimated prevalence in certain areas of 6.57 cases per 100,000 individuals [18]. Showing a mean disease duration of around 10 years, the onset usually occurs in the fourth decade, but can vary from childhood to late adulthood with an inverse correlation between age of onset and CAG repeat length [17]. Patients with SCA2 present with a cerebellar syndrome associated more often than SCA1 with saccadic slowing, hyporeflexia, tremor, or titubation. In some cases (especially those with a smaller number of CAG repeat expansions), SCA2 can present as parkinsonism [7]. First symptoms are slowly progressive gait ataxia and leg cramps, whereas dysarthria, kinetic, or postural tremor, decreased muscle tone, and tendon reflexes appear later [19]. Dystonia, chorea, and dementia have also been described as relatively common (almost 40% of the patients) [20]. Rarely, it can be associated with L-dopa-responsive parkinsonism [21–23], while ocular findings (i.e., nystagmus, slow saccadic eye movements, and supranuclear ophthalmoplegia) are more common [24].

**Fig. 2:**
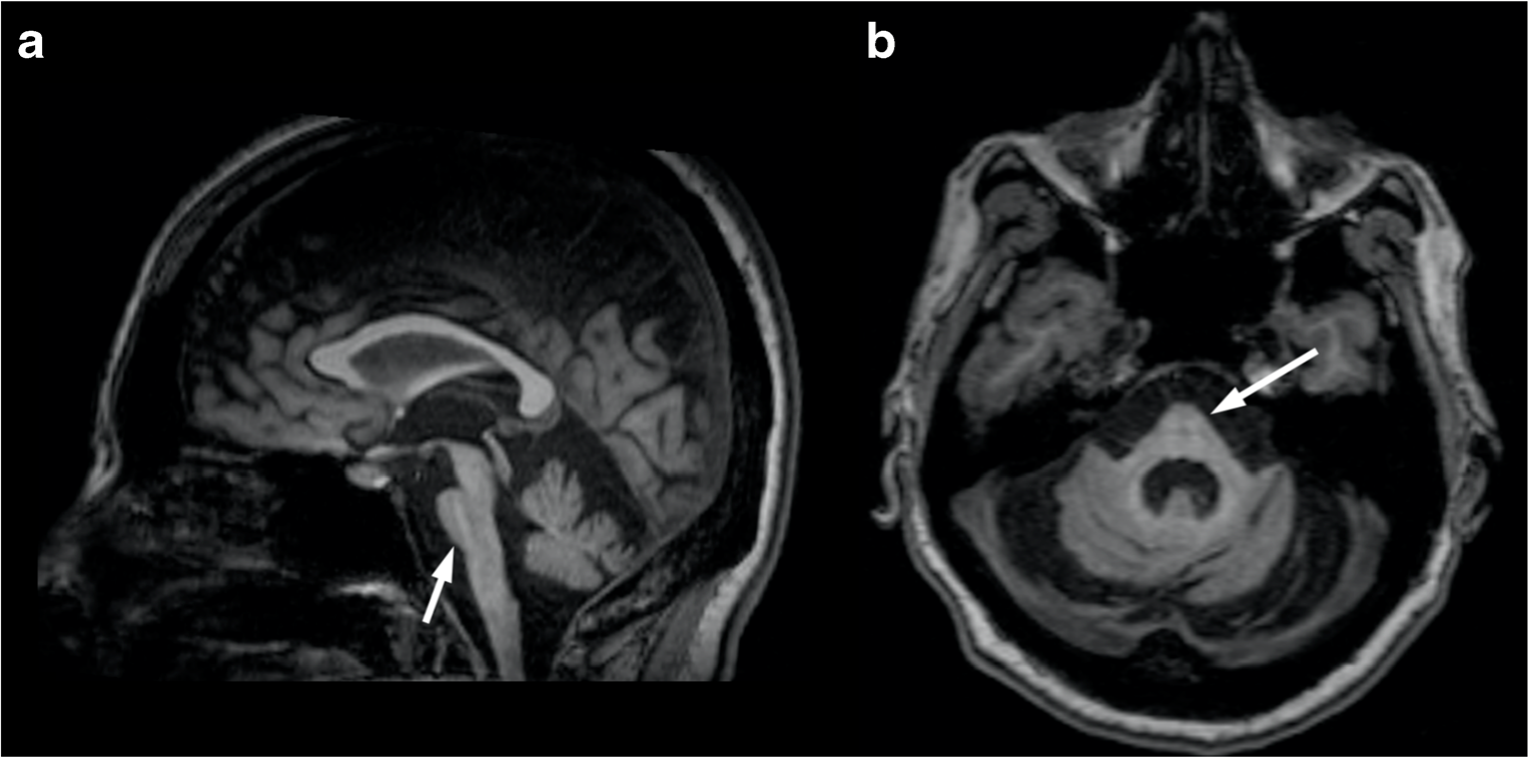
Imaging findings in a 38-year-old female SCA2 patient. Sagittal (**a**) and axial (**b**) multiplanar reconstructions of 3D T1-weighted volume show a global cerebellar volume loss, involving both the vermis and cerebellar hemispheres, along with significant brainstem atrophy, particularly affecting the pons which demonstrates a flattening of its inferior profile (arrows)

Brain MRI shows significant global atrophy of the cerebellum, with marked volume loss involving both hemispheres and the vermis. Furthermore, pontine atrophy (with flattening of the inferior part) (Fig. 2) and a variable degree of medulla oblongata and spinal cord volume loss are also usually depicted [18], with the severity of the olivo-ponto-cerebellar volume loss that correlates with clinical disability [25]. The “hot cross bun” sign may be present, resembling a sporadic multi-system atrophy pattern, while basal ganglia T2-hyperintensity are only rarely reported [25]. In advanced stages of the disease, a pattern of fronto-temporal atrophy with ventricular enlargement can also be depicted [26].

### Spinocerebellar ataxia type 3

Spinocerebellar ataxia type 3 (SCA3), also known as Machado-Joseph disease, is the most common SCA subtype worldwide and is caused by abnormal CAG trinucleotide repeats (52–86) in *ATXN3* gene encoding for the *Ataxin3* protein [9, 27]. Its onset usually occurs between the second and fifth decade, being characterized by ataxia, dysarthria, hyperreflexia, diplopia, and nystagmus [28]. Ambulation difficulty progressively increases, with assistive devices usually required 10 to 15 years after onset [28], while upper motor neuron signs are often present and may mimic hereditary spastic paraplegia [29, 30]. Sleep disturbance and impaired executive and emotional functioning have also been reported [31, 32]. The disease duration is variable from few up to 30 years after onset, with exitus usually occurring due to pulmonary complication and cachexia [33].

**Fig. 3:**
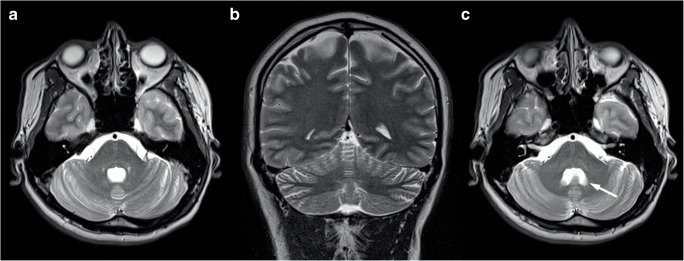
Axial (**a**, **c**) and coronal (**b**) T2-weighted images showing mild cerebellar atrophy, with enlargement of the 4th ventricle (arrow), in a 30-year-old female SCA3 patient

Brain imaging reveals a variable degree of ponto-cerebellar atrophy, less severe compared to the one found in SCA1 and SCA2 patients, mainly involving the vermis and dentate nuclei with subsequent enlargement of the fourth ventricle [34] (Fig. 3). Also, similar to what is reported in SCA1 and SCA2, pontine T2-weighted hyperintensities may be present in these patients, resembling in some cases the “hot cross bun” sign morphology [12]. As the disease progresses, frontal and temporal lobe atrophy can be observed [35], while abnormal pallidal linear hyperintensities on T2-weighted and FLAIR sequences have been sporadically reported [36].

### Spinocerebellar ataxia type 6

Spinocerebellar ataxia type 6 (SCA6), characterized by an abnormal CAG trinucleotide repeat expansion in *CACNA1A* gene [37], shows an estimated prevalence of about 0.02 on 100,000 individuals [38]. The onset can be extremely variable, ranging from 19 to 73 years (mean onset age = 43–52 years), with a relatively preserved lifespan [39, 40]. Clinically, SCA6 usually presents with a “pure” cerebellar ataxia, although a mild peripheral neuropathy (as well as bradykinesia, dystonia, and pyramidal signs) can also occur in some patients [41]. Ocular abnormalities are also quite common, as they have been described in 50% of patients, mainly consisting of diplopia and downbeat nystagmus [42–44].

**Fig. 4:**
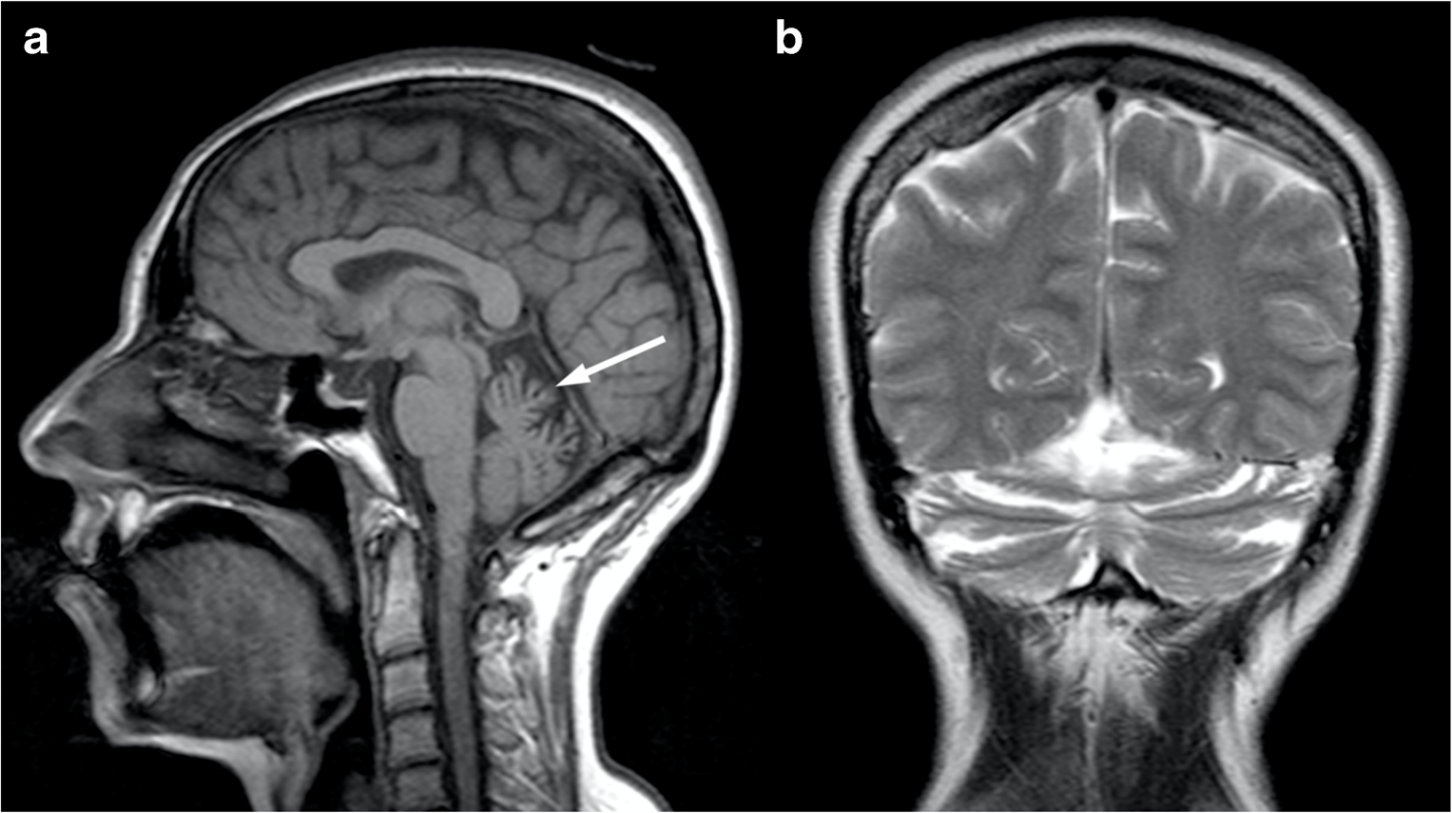
Brain MRI findings in a 56-year-old female SCA6. In the sagittal T1- (**a**) and coronal T2- (**b**) weighted images, it is appreciable a global cerebellar atrophy, with particular involvement of the vermis (arrow) and a relative sparing of the pons

At brain imaging, a variable degree of cerebellar atrophy has been described, involving the hemispheres but mostly the vermis (Fig. 4), with volume loss affecting the pons and middle cerebellar peduncles that has been only anecdotally reported in these patients [34]. No brain signal abnormalities have been reported in SCA6, nor supratentorial atrophy.

### Spinocerebellar ataxia type 7

Spinocerebellar ataxia type 7 (SCA7) shows a prevalence of less than 1:100,000, representing 2% of all SCAs [45, 46]. It is associated with the presence of more than 36 CAG trinucleotide repeat expansion in the *ATXN7* gene, although the development of symptoms has been also reported in patients with a lower number of repeats [47]. Disease onset ranges from childhood up to the sixth decade, with early-onset forms being more aggressive and rapidly progressive [48, 49]. Typical symptoms include cerebellar syndrome and visual loss caused by retinal degeneration, ultimately leading to complete blindness [50, 51].

**Fig. 5:**
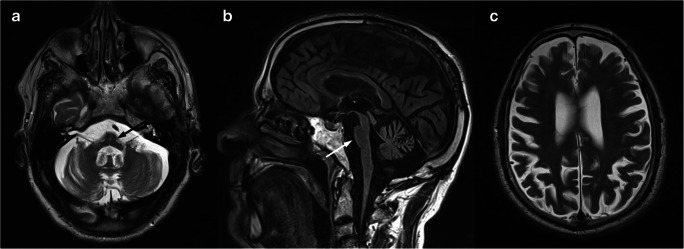
Neuroradiological findings in a 79-year-old male SCA7 patient. Axial T2- (**a**) and sagittal T1- (**b**) weighted sequences demonstrate a global cerebellar, as well as pontine (arrow), atrophy. Along with the infratentorial involvement, a diffuse supratentorial gray matter volume loss is also present (**c**)

MRI findings include cerebellar atrophy, mainly involving the superior part of the vermis, along with marked pontine atrophy [52] (Fig. 5), with the latter preceding and showing some degree of independence from the presence of cerebellar degeneration [53]. The “hot cross bun” sign has been reported in only one SCA7 patient [54], while no further reports describing signal alterations in SCA7 are available in literature. Finally, a variable degree of supratentorial atrophy can be present in these patients [52] (Fig. 5).

### Spinocerebellar ataxia type 8

Spinocerebellar ataxia type 8 (SCA8) accounts for 2–5% of autosomal dominant forms of inherited ataxia and is more common in Finland [55, 56]. It is caused by the abnormal expansion of both an expanded CTG trinucleotide repeat in the *ATXN8OS* gene and the complementary CAG repeat in the *ATXN8* gene [57]. The onset is extremely variable, ranging from 1 to 73 years, and the phenotype is characterized by ataxia, scanned dysarthria, and tremor, with reflex hyperactivity that may be present in severe cases [58]. Progression is usually independent of the age of onset and may take decades, though it does not significantly shorten lifespan [59, 60].

**Fig. 6:**
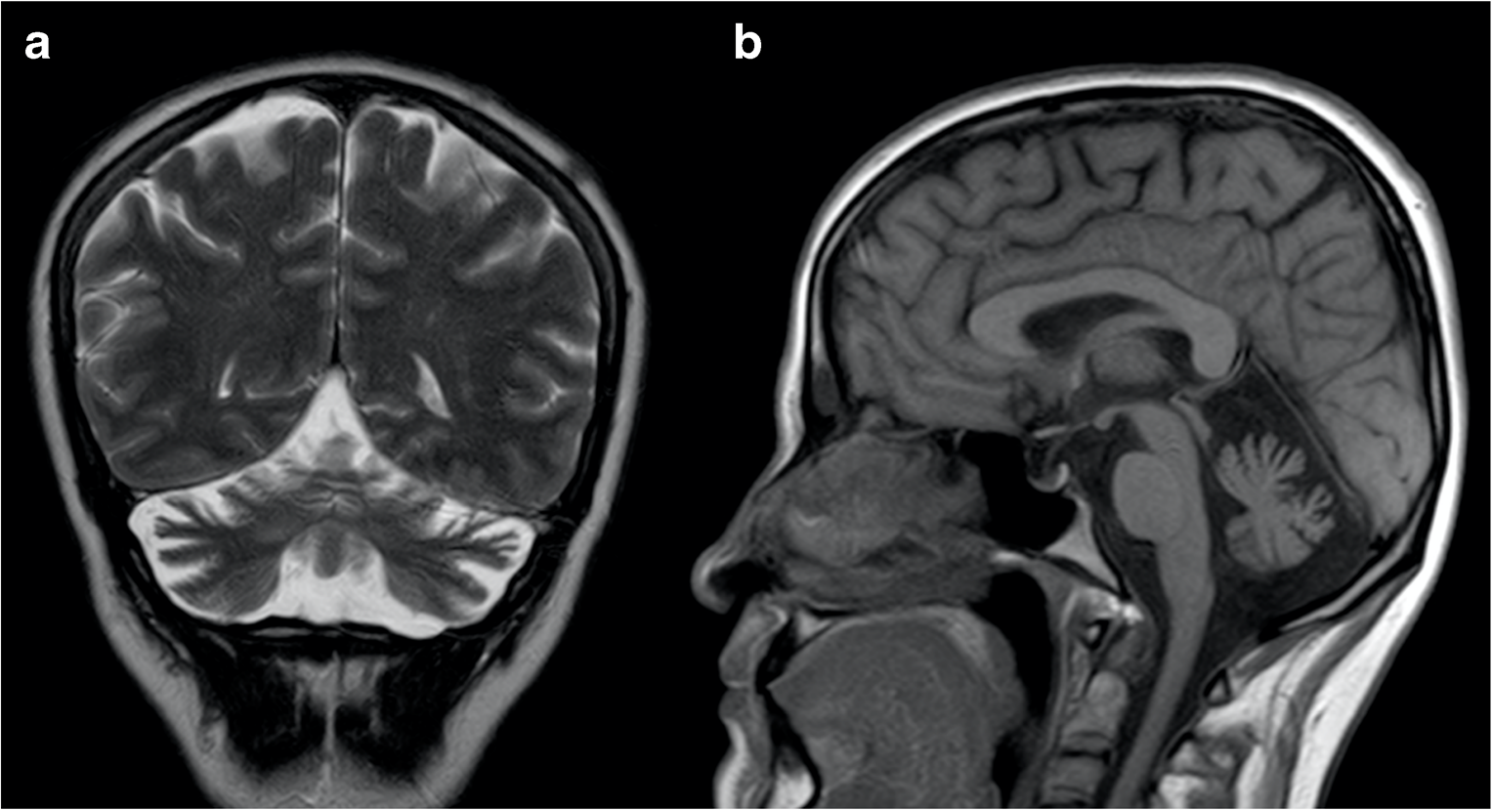
Brain MRI scan of 43-year-old male SCA8 patient. Coronal T2- (**a**) and sagittal T1- (**b**) weighted images show global cerebellar atrophy, with a relative sparing of the pons

On brain MRI, cerebellar atrophy affecting both the hemispheres and the vermis is usually found, with preservation of the brainstem and the cerebral hemispheres [61] (Fig. 6). No signal abnormalities have been described, except for a case of “hot cross bun” sign [54], while mild spinal cord atrophy can sometimes be present [58].

### Spinocerebellar ataxia type 17

Spinocerebellar ataxia type 17 (SCA17) is caused by the expansion of a CAG trinucleotide (42 or more repeats) in the TATA-box-binding protein gene (*TBP*) [62, 63]. Similarly to SCA8, it is also characterized by wide variability in its age of onset (from 3 to 75 years, mean = 34.6 years) [64]. The clinical features include cerebellar ataxia, cognitive decline, psychiatric symptoms, parkinsonism, and hyperkinetic disorders (e.g., chorea or dystonia, hence SCA17 being also referred to as Huntington disease-like 4 [65]). Among the abovementioned symptoms, ataxia and psychiatric abnormalities usually represent the initial manifestations of the disease, being then followed by involuntary movement, parkinsonism, dementia, and pyramidal signs [62, 66–68].

Conventional brain MRI shows a variable degree of cerebellar atrophy, affecting both the vermis and the hemispheres [25]. No significant changes are usually reported in the supratentorial areas, both in terms of signal changes or atrophy, with the exception of a single case showing a T2-hyperintense putaminal rim [69].

## Autosomal recessive ataxias

### Friedreich’s ataxia

Friedreich’s ataxia (FRDA) is the most common autosomal recessive ataxia, with an estimated prevalence in Europe between 1 in 750,000 (Finland) and 1 in 20,000 (Northern Spain) [70]. It is caused by biallelic GAA trinucleotide repeat expansions in intron 1 of the *FXN* gene on chromosome 9q21, encoding Frataxin [71]. FRDA first symptoms typically present between the age of 10 and 15, with onset after 25 and 40 years considered as late and very late onset, respectively [70, 72]. FRDA patients experience a shortened lifespan (average 35–40 years), with the most common cause of exitus represented by cardiac dysfunction [73]. From a neurological standpoint, FRDA is a disorder affecting both the central and peripheral nervous systems, with gait and limb ataxia, dysarthria, and lower limb areflexia that are present in almost all cases [74]. Pyramidal weakness is a relatively late sign, much more prominent in the lower limbs, while hearing difficulties due to acoustic neuropathy and eye movement abnormalities (e.g., square-wave jerks) are common signs of FRDA [74]. Systemic involvement is present in this condition, as shown by the presence of musculoskeletal (i.e., scoliosis and equinovarus deformities), cardiac (i.e., cardiomyopathy), and endocrine (i.e., diabetes mellitus) abnormalities [70, 74–76].

**Fig. 7:**
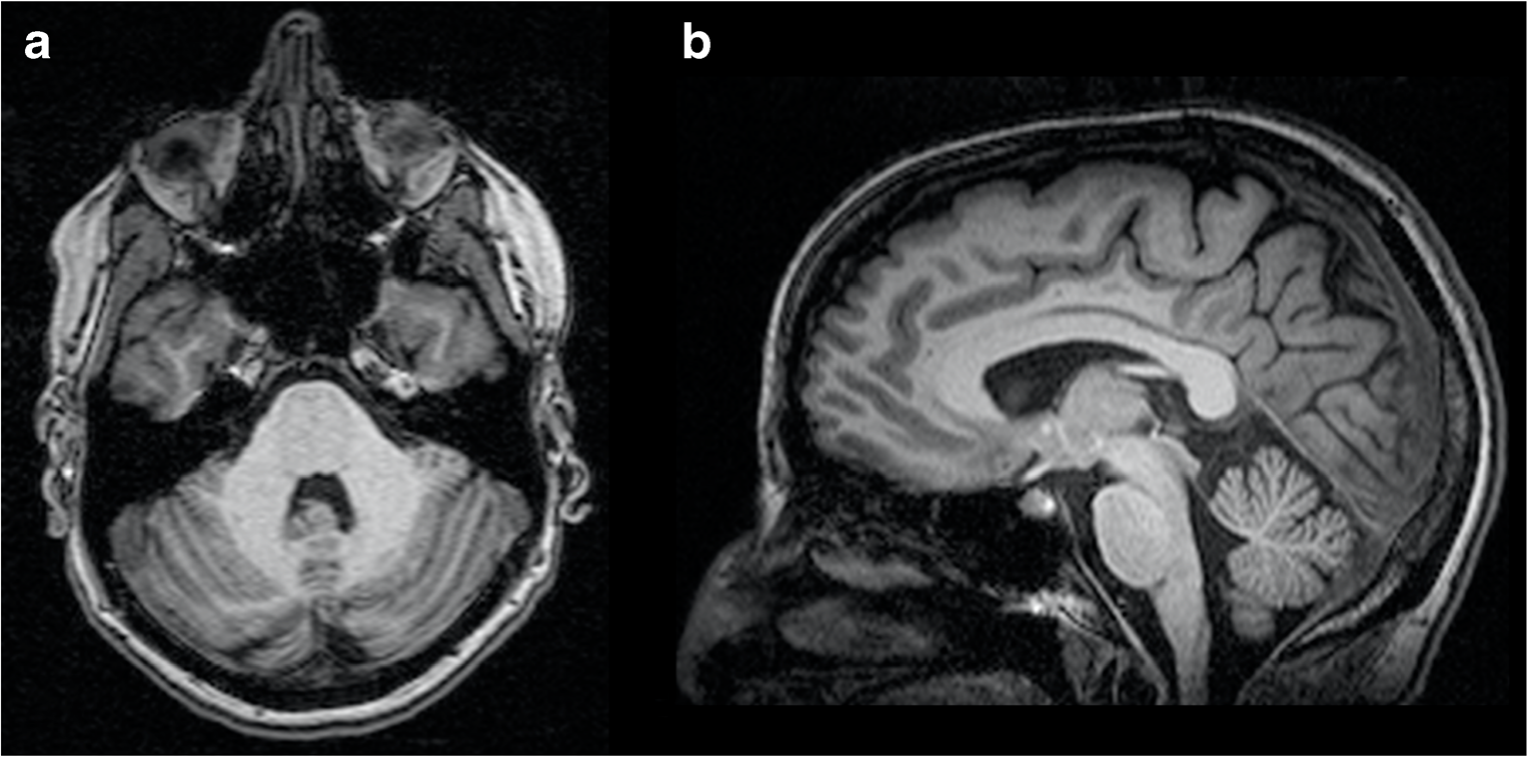
Axial (**a**) and sagittal (**b**) multiplanar reconstructions of a 3D T1-weighted volume show the typical brain MRI appearance in FRDA patients, as depicted in this 20-year-old male subject where a preserved cerebellar volume is present

Unlike most of the inherited ataxias, brain MRI in FRDA patients typically shows normal cerebellar volume (Fig. 7), or only mild atrophy of the upper portion of the vermis, while global cerebellar atrophy can be rarely found in very-late-onset patients [77, 78]. Indeed, the main imaging finding in this condition is a decrease of the antero-posterior diameter of the medulla oblongata and the cervical spinal cord, sometimes associated to signal abnormalities in the posterior or lateral columns [79], a finding consistent with degeneration of the ascending dorsal column system [80]. Furthermore, dentate nuclei atrophy, with increased iron accumulation, may be detected by susceptibility-weighted imaging [78, 81–83]. The supratentorial compartment is usually spared in this condition, both in terms of signal changes and volume loss.

### Autosomal recessive spastic ataxia of Charlevoix-Saguenay

Autosomal recessive spastic ataxia of Charlevoix-Saguenay (ARSACS) shows a high prevalence in northeastern Quebec Canada [84], although cases have been reported outside Canada in recent decades [85–87]. It is caused by autosomal recessive mutations in the *SACS* gene (13q11), which encodes a large protein named *Sacsin*, that might have roles in mitochondrial function, protein chaperoning, and the ubiquitin-proteasome system [88, 89]*.* The mean age at onset is approximately 6 years (range: 0–40 years), but an increasing number of cases with disease onset in teenage or early adult ages have been reported [90]. Disease progression is slow, and patients become wheelchair bound by the third or fourth decade of life. Most ARSACS patients show a typical clinical triad characterized by early-onset cerebellar ataxia, lower limb spasticity, and peripheral neuropathy [91]. Other symptoms and signs include dysarthria, nystagmus, and hypermyelination of the retinal fibers [91].

**Fig. 8:**
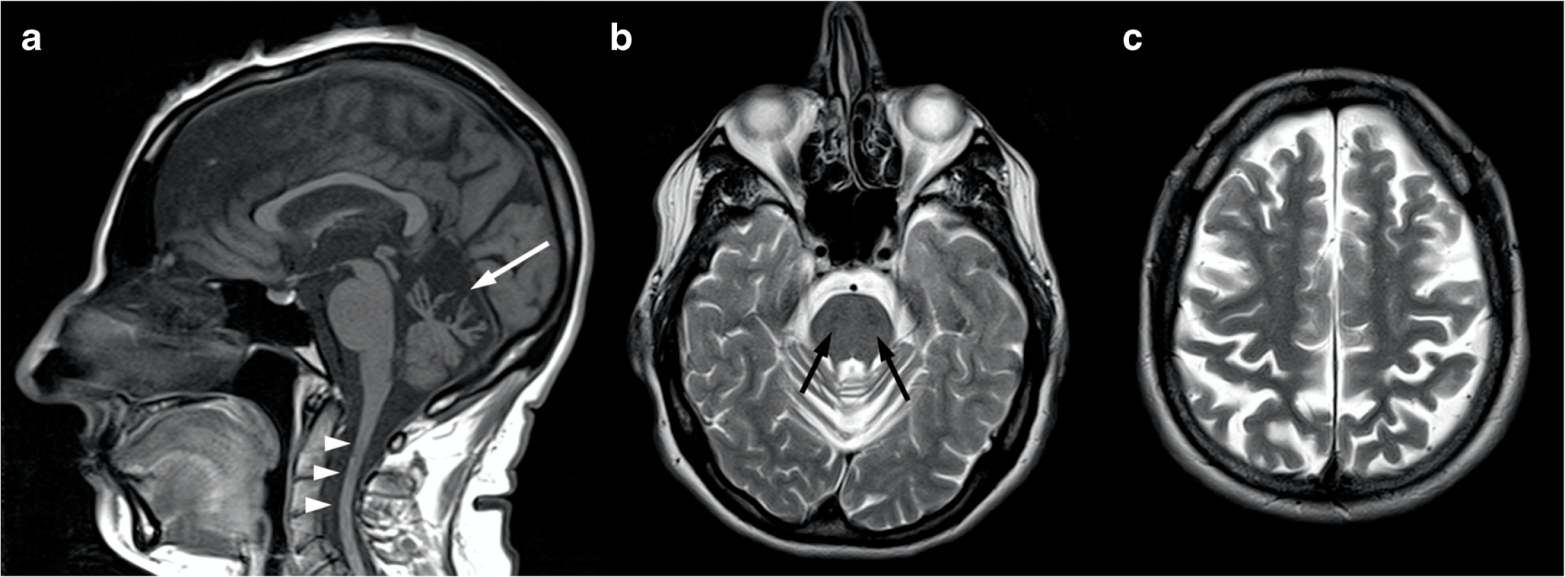
Brain MRI findings in a 45-year-old female ARSACS patient. A predominant superior vermian atrophy is shown on a sagittal T1-weighted sequence (**a**, white arrow), along with as a mild T2-weighted hypointensity affecting the transverse pontine fibers (**b**, black arrows). Parietal (**c**) and cervical spinal cord (**a**, arrowheads) atrophy is also evident

Brain MRI features include early and progressive superior vermis atrophy and linear hypointensities on T2-weighted images in the pons near the pyramidal tracts [92] (Fig. 8). Recent studies also found T2-hyperintensities of the lateral pons when merging into the middle cerebellar peduncles that appear thickened, probably related to abnormally large transverse ponto-cerebellar fibers, along with a frequent association with posterior fossa arachnoid cysts [93]. Furthermore, bilateral parietal atrophy and short-stretched thinning of the posterior mid-body of the corpus callosum can be depicted in ARSACS patients [93], as well as a thinning of the cervical spinal cord [94] (Fig. 8).

### Ataxia with oculomotor apraxia type 1

Ataxia with oculomotor apraxia type 1 (AOA1) is caused by biallelic mutations in the *APTX* gene at locus 9p13.3, which encodes for a nuclear histidine triad (HIT) protein, named *aprataxin* [95]. The first manifestation is usually represented by progressive gait imbalance (mean age of onset = 4.3 years; range = 2–10 years), with a mean disease duration of 29.8 ± 14.8 years [96]. The clinical phenotype of AOA1 is characterized by an early-onset cerebellar syndrome, nystagmus, dysarthria, oculomotor apraxia, areflexia, peripheral axonal neuropathy, muscle weakness, and a variable degree of intellectual disability [97]. Laboratory findings include hypoalbuminemia and hypercholesterolemia, while alpha-fetoprotein (AFP) value is usually relatively increased, although less than what was reported in AOA2 patients [98].

**Fig. 9:**
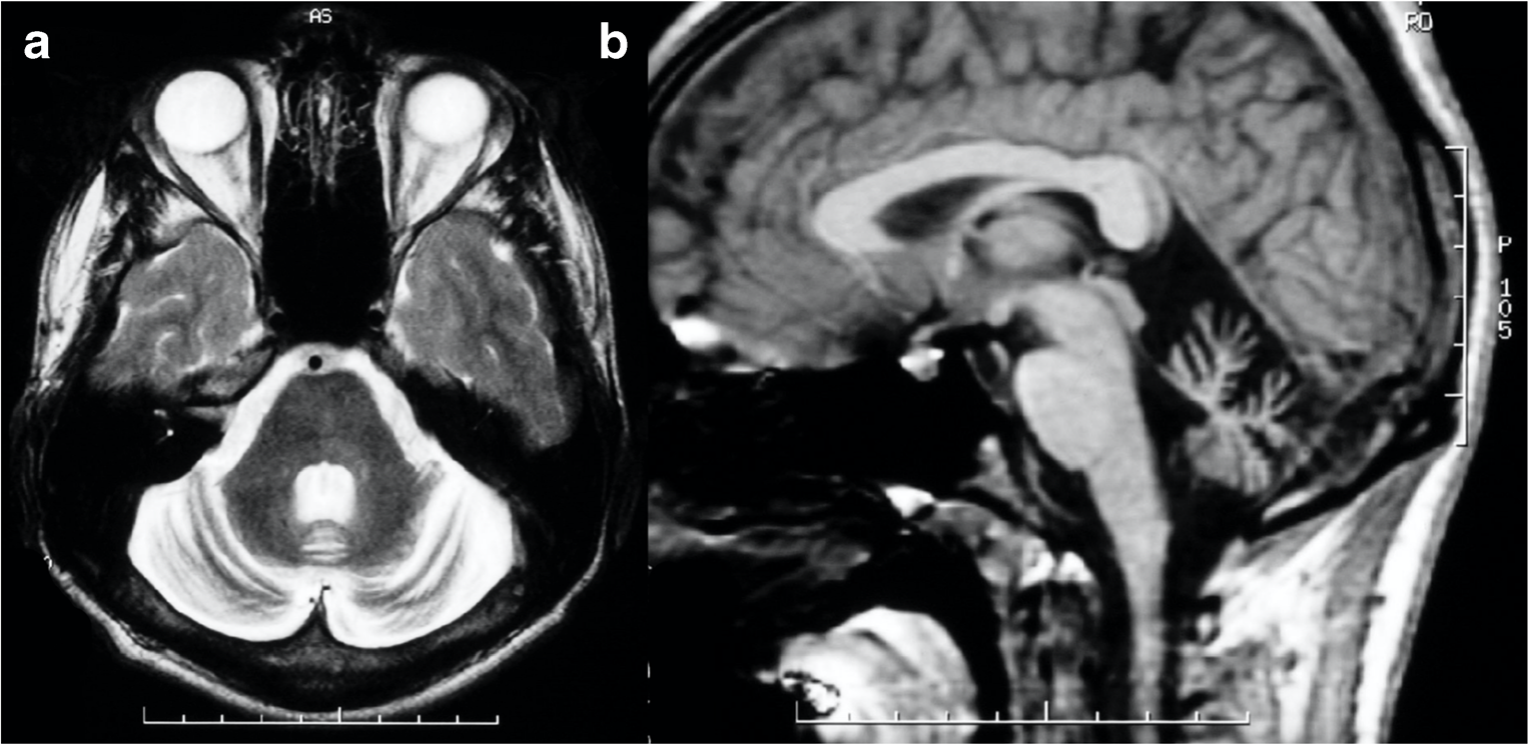
Axial T2- (**a**) and sagittal T1- (**b**) weighted images of an 18-year-old male AOA1 patient showing a severe global cerebellar atrophy, with relative sparing of the brainstem

MRI findings include diffuse cerebellar atrophy, mainly involving the anterior vermis, and possible brainstem atrophy, without T2-weighted signal changes [96] (Fig. 9). Disappearance of the dentate nuclei hypointensity on susceptibility-weighted imaging (SWI) has been described in AOA1 patients, along with a preserved volume, findings suggestive of an iron content change in this structure [99].

### Ataxia with oculomotor apraxia type 2

Ataxia with oculomotor apraxia type 2 (AOA2) is caused by mutations in the *senataxin* (SETX) gene at locus 9q34, which encodes for a protein suspected to be a DNA/RNA helicase [95, 100, 101]. It is a progressive, disabling cerebellar ataxia occurring within the second decade [102]. The clinical phenotype is characterized by progressive cerebellar ataxia, sensorimotor peripheral neuropathy, occasional oculomotor apraxia (relatively less frequently than the frequency reported in AOA1 patients), strabismus, chorea, and/or dystonia [102]. Laboratory examination reveals elevated AFP serum levels (more than those reported in AOA1 patients) and less frequently elevated creatine kinase serum level [98].

**Fig. 10:**
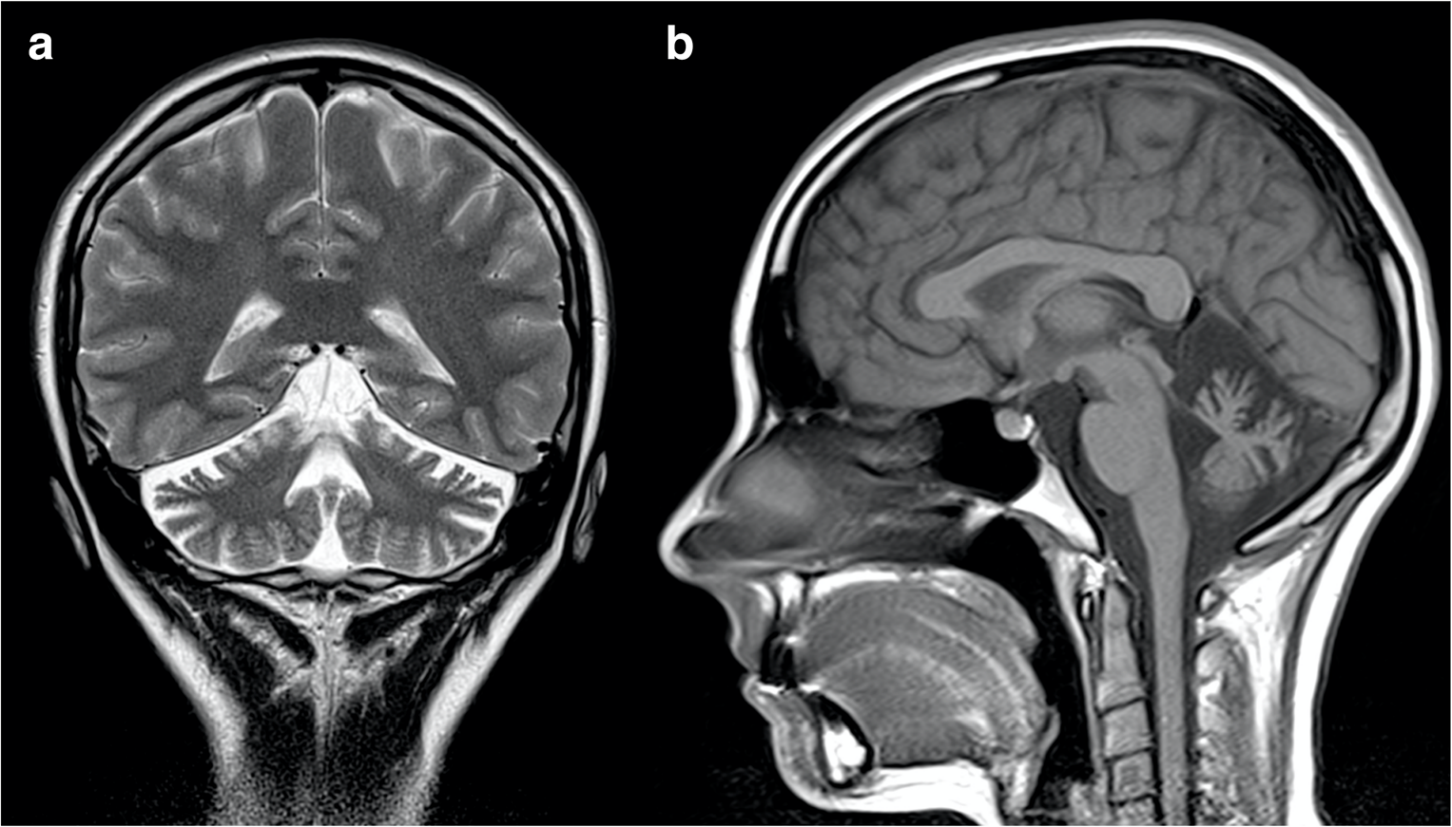
Coronal T2- (**a**) and sagittal T1- (**b**) weighted images of a 25-year-old female AOA2 patient showing a moderate global cerebellar and a relative brainstem sparing

From a neuroradiological standpoint, MRI findings are very similar to those found in AOA1 patients (Fig. 10), with cerebellar atrophy (with prominent involvement of the vermis and the anterior lobe) and loss of dentate SWI hypointensity and relative supratentorial sparing [99, 103].

### Spastic paraplegia 7

Spastic paraplegia 7 (SGP7) is the fourth cause of genetic ataxia in the UK, and the second most common cause of recessive ataxia (Kara2016). Indeed, recent studies demonstrated that SPG7 mutations are a frequent cause of undiagnosed cerebellar ataxias with adult-onset and pyramidal signs and provided the minimum prevalence of SPG7-related disease at 0.72/100,000 [104]. It appears to have a predilection for male patients (83%), with an average age of symptoms onset at 41.7 years [105]. Almost all patients show cerebellar ataxia at the diagnosis, usually along with mild spasticity and ocular findings [104]. Some patients present with a complicated phenotype of spastic paraplegia, associated with optic neuropathy, urinary urgency, scoliosis, pes cavus, neuropathy, and amyotrophy [106].

**Fig. 11:**
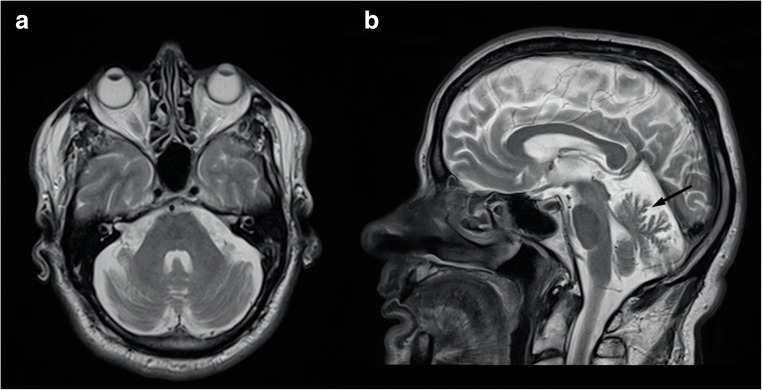
Imaging finding in a 69-year-old male SPG7 patient. Brain MRI axial (**a**) and sagittal T2- (**b**) weighted images show the presence of a mild global cerebellar atrophy, with particular involvement of the vermis (black arrow)

At the brain MRI examination, the most frequent feature is represented by mild cerebellar atrophy, mostly involving the vermis [105] (Fig. 11). An increased T2-weighted signal in dentate nuclei is reported in this condition, while the red nucleus signal is usually normal [105]. No supratentorial involvement has been reported so far in SPG7 patients.

### Ataxia-telangiectasia

Ataxia-telangiectasia (AT) is a primary immunodeficiency disease caused by mutations in AT mutated (*ATM*) gene encoding a serine/threonine protein kinase [107, 108]. Disease onset is usually between 6 and 18 months, and the clinical phenotype can be highly variable, including progressive cerebellar ataxia, oculo-cutaneous telangiectasia, variable immunodeficiency, radiosensitivity, susceptibility to malignancies, and metabolic disorders [109]. Involuntary movements (e.g., chorea, dystonia, athetosis, myoclonic jerks, or various tremors) can be present, while cognitive impairment is usually observed in up to 30% of patients [109]. Disease duration is less than 25 years, with the two most common causes of exitus that are chronic pulmonary diseases and malignancy [110].

**Fig. 12:**
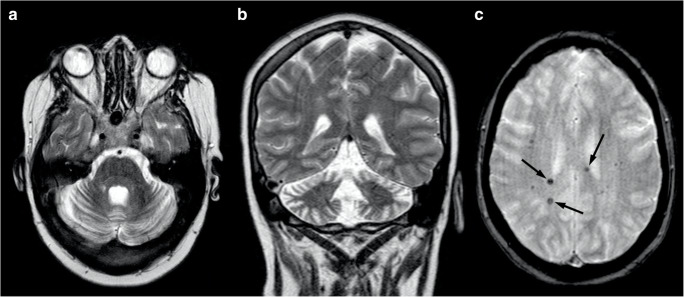
Neuroradiological findings in a 26-year-old female AT patient. A mild global cerebellar atrophy is shown in both axial (**a**) and coronal (**b**) T2-weighted images, while the T2*-weighted sequence allows for the depiction of small punctuate hypointense foci (arrows in **c**) representing both hemosiderin deposits and telangiectatic vessels

At brain MRI, AT patients typically demonstrates progressive cerebellar atrophy, with significant vermian involvement [111] (Fig. 12). In addition, in some cases, supratentorial white matter T2-weighted and SWI hypointensities can be detected, representing hemosiderin deposits and deep cerebral telangiectatic vessels [111, 112] (Fig. 12). In older patients, diffuse T2-weighted hyperintensity of the cerebral white matter can be found as an expression of vascular damage [112].

### Ataxia with isolated vitamin E deficiency

Ataxia with isolated vitamin E deficiency (AVED) is caused by mutations in the alpha tocopherol transfer protein (*TTPA*) gene, mapped on chromosome 8q13 that codifies for a protein which binds alpha tocopherol and very-low-density lipoproteins (VLDLs) in the liver [113]. The mutated protein impairs the incorporation of vitamin E into plasma VLDL, with subsequent systemic oxidative stress damage [114].

The age of onset is variable, from early childhood to very late adult life [115]. The neurological phenotype is very similar to the one found in FRDA patients (progressive cerebellar ataxia with posterior column involvement, Romberg’s sign, and pyramidal spasticity) [116], although AVED patients are more frequently experiencing head titubation and dystonia, with less pronounced cardiovascular impairment and neuropathy [117]. In the presence of this phenotype, the evidence of very low serum vitamin E levels, in the absence of fat malabsorption, is highly suggestive of AVED [118].

Similarly to the neurological phenotype, also brain MRI findings in AVED patients resemble those found in FRDA, with the presence of preserved cerebellar volumes in almost all cases [119], although mild hemispheric atrophy has been reported in some subjects [120]. Finally, no cervical spine abnormalities are usually reported in AVED patients.

### Cerebrotendinous xanthomatosis (CTX)

Cerebrotendinous **x**anthomatosis CTX is a neurometabolic storage disorder caused by mutations in the *CYP27A1* gene, mapped on chromosome 2q33 and codifying for the 27-hydroxylase, with mutations that lead to reduced enzymatic activity and elevated levels of cholestanol, cholesterol, and bile alcohols [121]. The age of onset may range from infancy to adulthood, with a median age of clinical presentation that ranges between 9 and 19 years, although diagnosis usually occurs only later during adulthood [122].

Clinical symptoms and signs are both neurological and non-neurological. Among systemic manifestations, xanthomas present early in childhood and enlarge over time, with the Achilles tendon being the most common affected site, although they can also be found in other subcutaneous tissues, especially at the level of the elbow [123]. Other non-neurological manifestations include neonatal cholestatic jaundice, chronic diarrhea, and ocular manifestations (e.g., cataract, optic neuropathy, optic disk paleness, and premature retinal senescence) [124]. Hallmark neurological signs are represented by intellectual disability, pyramidal signs (i.e., spasticity, hyperreflexia, extensor plantar responses), cerebellar signs (ataxia, dysarthria, nystagmus), and peripheral neuropathy, while other symptoms include epileptic seizures and parkinsonism [122].

**Fig. 13:**
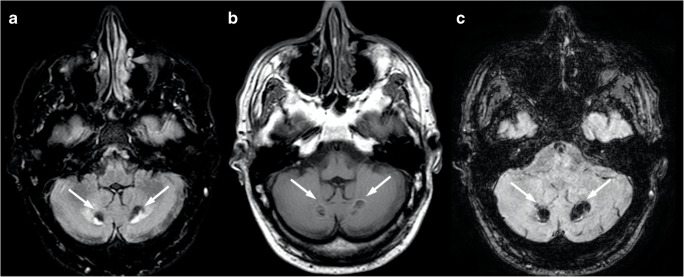
Brain MRI findings in a 42-year-old male CTX patient. Along with a mild global cerebellar atrophy, it is present gliosis and calcifications of the deep cerebellar WM, extending to the peri-dentate region (arrows), as shown in the axial FLAIR (**a**), T1-weighted (**b**), and SWI (**c**) sequences

MRI studies show, along with a variable degree of cerebral and cerebellar atrophy, focal or diffuse subcortical and periventricular white matter T2-weighted hyperintensities, sometimes also found affecting the midbrain [125]. Areas of low T2-weighted signal can also be observed, referable to vacuolation and calcifications, and have been proved to be a possible biomarker of disease progression [125]. Furthermore, a non-homogeneous T2-weighted hyperintense signal in dentate nuclei and surrounding cerebellar white matter has been demonstrated in most of the CTX patients, apparently showing some degree of correlation with clinical severity [125] (Fig. 13).

### Autosomal recessive spinocerebellar ataxia type 10

Autosomal recessive spinocerebellar ataxia type 10 (SCAR10), also known as autosomal recessive cerebellar ataxia type 3 (ARCA3), is caused by homozygous or compound heterozygous mutations in the anoctamin 10 (*ANO10*) gene, mapped on 3p21.33 [126].

The onset is reported in the teenage or young adult years [127], usually presenting with gait and limb ataxia, dysarthria, nystagmus, and occasional involvement of lower motor neurons, while the cognitive status may be normal or impaired [128]. A characteristic finding of this condition is the presence of low levels of coenzyme Q10 (CoQ10) [129].

**Fig. 14:**
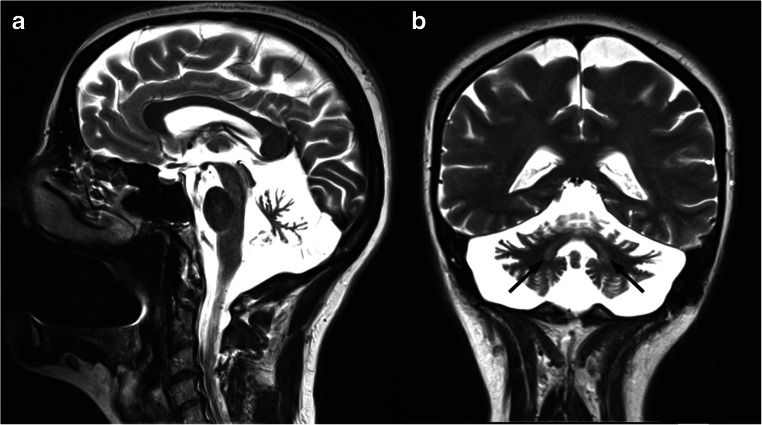
A 36-year-old female SCAR10 patient showing a global cerebellar atrophy, along with a mild T2-weighted hyperintensity affecting both dentate nuclei (black arrows in **b**)

At the brain MRI scan, SCAR10 patients show moderate to marked cerebellar atrophy [129–131], in some cases coupled to a mild T2-weighted hyperintensity of the DN [130, 131] (Fig. 14). Diffuse supratentorial cortical atrophy, more pronounced in the fronto-parietal regions, can be also detected in older patients [129, 131].

## X-linked ataxias

### Fragile X-associated tremor/ataxia syndrome

FXTAS constitutes a progressive neurodegenerative movement disorder caused by a fragile X “premutation,” defined as 55–200 CGG repeats in the 50-untranslated region of the *FMR1* gene, while the presence of more than 200 repeats results in the development of the fragile X syndrome (a heritable form of cognitive impairment) [132].

FXTAS is more common in males than in females, the onset is usually over the age of 50 [133], with a life expectancy that ranges between 5 and 25 years [134]. Classical neurological manifestations include kinetic tremor and cerebellar ataxia, but cognitive decline, psychiatric disorders, peripheral neuropathy, and autonomic dysfunction are also frequently described [135, 136].

**Fig. 15:**
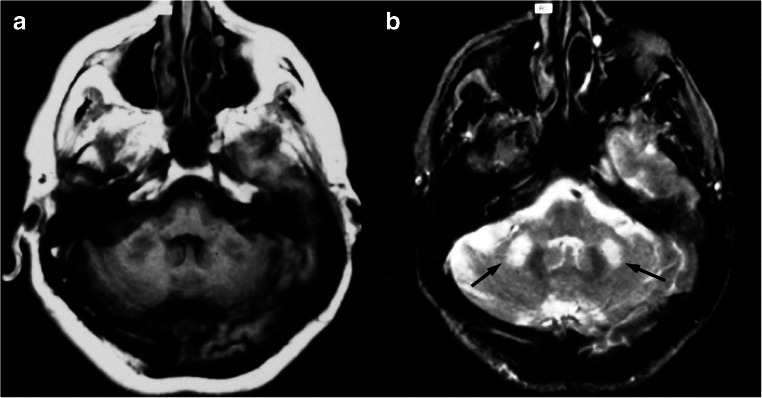
Imaging findings in a 55-year-old male patient with FXTAS. Axial T1- (**a**) and T2- (**b**) weighted sequences show the presence of a mild global cerebellar atrophy, associated with the typical T2-weighted hyperintensity of the middle cerebellar peduncles (black arrows in **b**)

At brain MRI scan, FXTAS patients show characteristic features, useful for a correct diagnosis along with clinical signs and genetic tests. Indeed, the revised FXTAS diagnostic criteria [137] include the presence of two major radiological features, namely the presence of white matter lesions in middle cerebellar peduncles and in corpus callosum splenium. The first finding is considered a radiological hallmark of this condition (Fig. 15), although also being reported in cerebellar type multiple system atrophy (MSA-C) or acquired hepato-cerebral degeneration [86], while the second has been recently reported as a more reliable MRI sign [134]. Other common and minor findings include the presence of T2-weighted hyperintensities in the pons (i.e., the “hot cross bun” sign) and in the afferent projections of the middle and superior cerebellar peduncles, as well as supratentorial areas (e.g., insula or periventricular white matter), along with a generalized brain, brainstem, and cerebellar atrophy [138].

## Conclusion

Neuroradiological diagnosis of hereditary degenerative ataxias can be very challenging, given that usually brain MRI scans show, in most of these conditions, the presence of non-specific and sometimes overlapping imaging findings.

In this work, we have reviewed the main clinical and conventional imaging findings of the most common hereditary degenerative ataxias. A proper assessment of imaging and clinical data is crucial for the neuroradiologist to identify some and exclude other conditions, leading the clinician to a more appropriate genetic testing to ultimately achieve a diagnosis.
